# Ameloblastoma: An Updated Narrative Review of an Enigmatic Tumor

**DOI:** 10.7759/cureus.27734

**Published:** 2022-08-06

**Authors:** Suhani Ghai

**Affiliations:** 1 Surgical Oncology, Dharamshila Narayana Superspeciality Hospital, New Delhi, IND

**Keywords:** adamantinoma, multicystic ameloblastoma, brafv600e mutation, ameloblastoma, maxillary neoplasms, maxilla, mandibular neoplasms, mandible, odontogenic tumors, jaw neoplasms

## Abstract

Ameloblastoma is one of the most common benign odontogenic tumors of the jaw that constitutes about 10% of all tumors that arise in the mandible and maxilla. It is a slow-growing but locally invasive tumor that presents with painless swelling of the mandible or maxilla. The World Health Organization (WHO) classification of 2017 describes ameloblastomas of the following four types: ameloblastoma; unicystic ameloblastoma; extraosseous/peripheral ameloblastoma; and metastasizing ameloblastoma. The diagnosis of ameloblastoma requires computerized tomography (CT) imaging as well as a biopsy. A biopsy is helpful in differentiating ameloblastoma from ossifying fibroma, osteomyelitis, giant cell tumor, cystic fibrous dysplasia, myeloma, and sarcoma. The best treatment of ameloblastoma is aggressive en bloc resection with simultaneous reconstruction. The high recurrence rate and large tissue defects have been long-standing issues in the treatment of ameloblastoma. Recent molecular developments strongly suggest the possibility of targeted therapy with better outcomes in ameloblastomas. We present a detailed updated narrative review of our current understanding and management of this enigmatic tumor.

## Introduction and background

Ameloblastoma is a common but enigmatic tumor of the jaw and constitutes about 10% of all tumors that arise in the mandible and maxilla [1]. It is one of the most common benign odontogenic tumors. Its enigmatic nature is manifested by its slow-growing rate, similar to benign tumors, but having the characteristics of being locally invasive, with a high recurrence rate and a metastatic potential similar to malignant tumors. Here we review our current understanding and management of this enigmatic tumor.

## Review

Methods

An electronic literature search in PubMed, EMBASE, and Google Scholar was performed for this review using the search terms ‘ameloblastoma’ and ‘odontogenic tumor’. The inclusion criteria were all types of articles published in PubMed and related only to humans. The exclusion criteria were articles for which full text was not available, were not in English, or were gray literature. The focus of the searched literature was the definition, classification, epidemiology, clinical features, investigations, and treatment of ameloblastoma. From the articles retrieved in the first round of search, additional references were identified by a manual search among the cited references. The last literature search was performed on January 6, 2022.

Definition

Ameloblastoma is a locally invasive, slow-growing tumor of odontogenic epithelium, mainly arising from enamel tissue that has not undergone differentiation [1]. It was first recognized by Cusack in 1827 [2] and designated as an ‘adamantinoma’ in 1885 by the French physician Louis-Charles Malassez [3]. In 1930, it was rechristened ‘ameloblastoma’ by Ivey and Churchill [4]. In 2017, the World Health Organization (WHO) described ameloblastoma as one of the benign epithelial odontogenic tumors [5]. Mutations in genes that belong to the MAPK pathway are found in many ameloblastomas, the most common being the BRAFV600E mutation [6].

Classification

The WHO, in 1971, had included odontogenic tumors in its first histological classification and provided the clinicopathological criteria necessary for diagnosis. The fourth edition of the WHO Classification of Head and Neck Tumors was published in 2017 [5]. This latest classification is simplified from the previous versions and describes ameloblastomas of the following four types: ameloblastoma; unicystic ameloblastoma; extraosseous/peripheral ameloblastoma; and metastasizing ameloblastoma (Table 1) [5]. The adjective ‘solid/multicystic’ for conventional ameloblastoma was removed because it has no prognostic significance and can lead to confusion with unicystic ameloblastoma [7]. Desmoplastic ameloblastoma, which had been sub-categorized under ameloblastoma in the previous 2005 WHO classification, was also removed, as it was considered merely a histological variant of conventional ameloblastoma, despite its unique clinical and sometimes radiographic features [7]. Similarly, odontoameloblastoma, from the 2005 classification, has also been removed and is now more accurately described as ameloblastoma arising in an odontoma and not odontoameloblastoma. Metastasizing ameloblastoma was reclassified as a benign tumor rather than a malignant odontogenic tumor, as this tumor shows benign histopathology, in spite of its metastatic potential, thus rendering it difficult to differentiate histopathologically from conventional ameloblastoma [5].

**Table 1:** The classification of ameloblastoma according to the WHO Classification of Head and Neck Tumors, published in 2017

Benign epithelial odontogenic tumors	Frequency	Histological variants
Ameloblastoma	91%	Follicular, Plexiform, Acanthomatous, Granular Cell, Basal Cell, Keratopapillary, Desmoplastic
Ameloblastoma, unicystic type	6%	Luminal, Mural
Ameloblastoma, extraosseous/peripheral type	2%	
Metastasizing ameloblastoma	1%	

Recently, there has been an effort for the inclusion of adenoid ameloblastoma as a sub-type of ameloblastoma in the next revision of the WHO odontogenic tumor classification [8]. Adenoid ameloblastoma is a hybrid odontogenic tumor showing histopathological features of both ameloblastoma and adenomatoid odontogenic tumor. Adenoid ameloblastoma is demographically similar to conventional ameloblastoma but with histopathological differences and presents with a higher rate/multiple recurrences, indicating its biological aggressiveness [8].

Epidemiology of ameloblastoma

Ameloblastoma shows a variable geographic prevalence with a global incidence of 0.92 cases per million person-years [9]. Most epidemiological studies have revealed that ameloblastoma is either the most common or the second most common benign odontogenic tumor. In a Brazilian study of 6231 oral lesions, 185 (3%) were odontogenic tumors, all benign. Of these, the most frequent lesions were ameloblastomas (29%), followed by keratocystic odontogenic tumors (28%) and odontomas (19%) [10]. Similarly, two large series on odontogenic tumors from China, one comprising 759 cases and based on the 1992 WHO classification [11] and the other comprising 1642 cases and based on the 2005 WHO classification [12], had revealed that ameloblastoma was the most frequent odontogenic tumor (59% and 40%, respectively). However, in another Chinese series of 1309 odontogenic tumors, the most frequent was the keratocystic odontogenic tumor (39%), followed by ameloblastoma (37%) and odontomas (6%) [13]. In the United States and Canada also, ameloblastoma is the second most common odontogenic tumor next to odontoma [14-15].

Among the Indian studies, the largest study was carried out in the Marathwada region of Maharashtra, where the histopathology records from 1992 to 2012 were reviewed. Of the 125 benign odontogenic tumors, the most common was the keratocystic odontogenic tumor (45%) followed by ameloblastoma (35%), odontoma (7%), and adenomatoid odontogenic tumor (5%) [16].

The most common age of presentation of ameloblastoma is the 30-60 years age group with a slight male preponderance, and the most common site being mandible [17]. In a Chinese study by Lu et al., the mean age of presentation of ameloblastoma was about 31 years, with 91% of the tumors occurring in the mandible [11]. The male: female ratio was 1.5:1. Hatada et al. from Japan documented a mean age of 35 years with a male: female ratio of 1.6:1, with about 93% of tumors located in the mandible [18]. In a study on 52 cases of ameloblastomas from India, the predominant age of presentation was the third to fourth decades with a male preponderance; and the most common site was the lower jaw, especially the posterior aspect, both for new and recurrent cases [19]. In a study from the Marathwada region of India also, the age distribution showed a peak occurrence of the odontogenic tumor in the fourth decade (31%) [16]. Ameloblastoma in children is considered rare and in an Indian series out of 256 diagnosed cases of ameloblastoma, only 15% occurred in the pediatric age group with a male: female ratio of 2:1 [20].

This racial differentiation in the relative prevalence of ameloblastoma was elegantly brought out in a systemic review, which documented that the frequency of ameloblastoma was significantly higher in Asian and African hospitals compared to European and American hospitals [21].

Clinical presentation

Slow-growing, painless swelling of the mandible or maxilla is the most common presentation of ameloblastoma. Occasionally, ameloblastoma is detected incidentally on radiographs taken for other reasons [22]. Most cases of ameloblastoma (80%) occur in the mandible, predominantly in the posterior mandibular region. Maxillary ameloblastoma also mostly occurs in the posterior molar region [23]. An un-erupted third molar teeth can also be associated with ameloblastoma. The desmoplastic variant of ameloblastoma often occurs in the anterior part of the maxilla or mandible. The growth of ameloblastoma occurs in the buccolingual direction, resulting in significant expansion. The mean size of ameloblastomas at presentation is approximately 4 cm [24]. Pain is an uncommon symptom of ameloblastoma but can occur because of hemorrhage inside or adjacent to the tumor. Malocclusion, facial deformity, soft tissue invasion, or loosening of teeth are other signs and symptoms of ameloblastoma [25].

The unicystic variant of ameloblastoma presents most commonly in the pediatric age group. It possibly arises from a pre-existing dentigerous cyst or from a dental follicle because of its frequent dentigerous relationship with an un-erupted tooth. Thus, the third molar region is the most frequent location of unicystic ameloblastoma [26]. The peripheral variant of ameloblastoma most commonly presents as a slow-growing painless, gingival swelling in adults.

The mean age of occurrence of metastasizing ameloblastoma is 43±16 years, with a slight male predilection. The mandibular cases showed more metastasis than the maxillary cases, and lungs, followed by lymph nodes, are the most commonly affected secondary sites [27].

Ameloblastoma is a slow-growing tumor. A systematic review of 16 studies calculated the mean specific growth rate of ameloblastoma as 87.84% per year. The highest growth rate was associated with a solid, multicystic type of ameloblastoma while the lowest growth rate was associated with peripheral ameloblastomas [28].

Diagnosis

The diagnosis of ameloblastoma requires imaging (usually a CT scan) as well as a biopsy. The CT scan most commonly shows a well-defined, uni- or multilocular radiolucent expansile lesion. It is also good for the evaluation of any cortical destruction or soft tissue extension [29]. Although ameloblastoma originating within bone is often detected on dental X-rays (orthopantomogram, OPG) or plain film, however, the extent of soft tissue or bone invasion is often not accurately documented. On X-ray, the unicystic ameloblastoma appears as a lytic lesion with scalloped margins. It also shows impacted molars and resorption of tooth roots. The commoner multilocular ameloblastoma appears as the classic ‘‘soap bubble’’ appearance on an X-ray. For ameloblastoma arising from the maxilla, an MRI is more useful than CT, as it better characterizes any extension to the skull base, orbit, or paranasal sinuses. MRI is also the imaging modality of choice for desmoplastic ameloblastoma since it has ill-defined soft tissue borders that can be misdiagnosed as a fibro-osseous lesion [29-30]. The unicystic ameloblastoma is usually diagnosed only after a histopathologic examination because it appears like an odontogenic cyst both clinically and radiologically. On imaging modalities such as CT or MRI, the unicystic ameloblastoma has the appearance of a thinly corticated unilocular radiolucency usually in association with an un-erupted tooth and often leading to jaw expansion [26]. In the case of metastasizing ameloblastoma, a positron emission tomography (PET) scan is generally preferred to detect distant metastasis.

Since none of the radiological features are pathognomonic, the definite diagnosis of ameloblastoma is only established by biopsy. Histopathology helps in differentiating ameloblastoma from ossifying fibroma, osteomyelitis, giant cell tumor, cystic fibrous dysplasia, myeloma, and sarcoma. Preoperative staging in cases of malignant ameloblastoma is also established by biopsy [29,31]. Trucut needle biopsy can be obtained under CT guidance via a dental socket or a window of cortical erosion. For an incisional biopsy, disruption of the mucosa is required, which will ultimately need to be removed at surgery. Peripheral ameloblastoma can be easily biopsied since it is not covered by bone [29].

Histopathological findings

Histologically, ameloblastoma contains two types of cells: peripherally situated ‘basal cells’ that resemble ameloblasts and centrally situated suprabasal ‘epithelial cells’ that resemble stellate reticulum. The basal cells are hyperchromatic, columnar in shape with a palisaded arrangement, vacuolated cytoplasm, and with nuclei displaced away from the basement membrane (reversal of polarity). The epithelial cells have a bland cytological appearance with sparse mitotic figures in keeping with their slow rate of growth [26].

**Figure 1:**
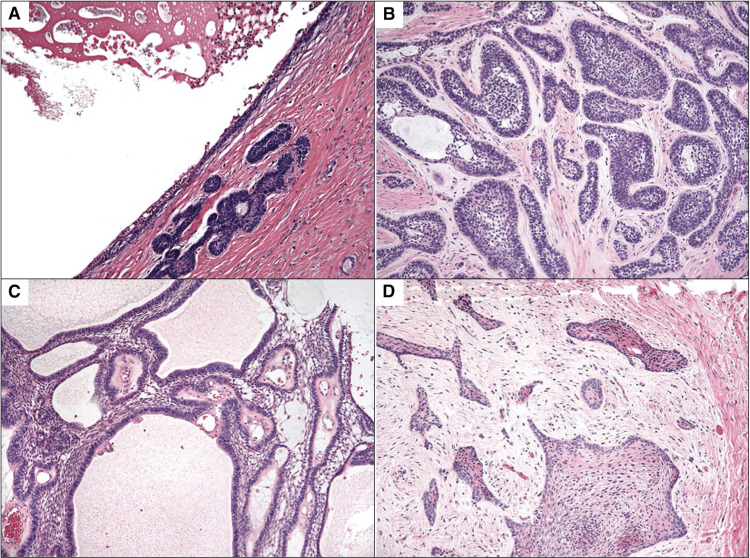
Figure depicting four histologic subtypes of ameloblastoma. A: unicystic, B: follicular, C: plexiform, and D: desmoplastic Reproduced with permission from McClary AC, West RB, McClary AC, et al. Ameloblastoma: a clinical review and trends in management. Eur Arch Oto-Rhino-Laryngol. 2016;273(7):1649-1661

In classical ameloblastoma (previously labeled as solid/multicystic ameloblastoma), these basal and epithelial cells, in turn, are arranged in two characteristic patterns: follicular and plexiform (Figure 1). In the follicular pattern, the epithelial cells are arranged in islands or follicles surrounded by connective tissue; while in the plexiform pattern, the epithelial cells are arranged in an interlacing plexiform network surrounding the connective tissue. Occasionally, ameloblastomas can show both these patterns in various proportions in the same tumor. Various other histological variants of multicystic ameloblastomas have been described, such as desmoplastic, acanthomatous, basal cell, granular cell, and keratopapillary ameloblastomas. These variants are often superimposed on one of the two main characteristic patterns and none of these variants reflects any difference in tumor behavior, except the desmoplastic ameloblastoma, which may be more aggressive [26].

The diagnosis of unicystic ameloblastoma is often challenging for the pathologist, as classical diagnostic features are often not visible. Solid tissue is generally absent and only membranous pieces of cyst wall are recovered. The basal cells of the cyst wall show limited elongation, and the characteristic nuclear palisading is usually limited to only a small group of cells. Although the suprabasal cells may stratify in the form of stellate reticulum, they may have superimposed inflammation. The unicystic ameloblastoma is of two histological variants: luminal and mural. In the luminal variant, the wall of the cyst appears as a uniform sac that is lined by the ameloblastoma epithelium. Sometimes, the wall thickenings composed of ameloblastoma cells invaginate into the lumen. In the mural variant of ameloblastoma, the tumor islands infiltrate the fibrous wall much like the conventional ameloblastoma [26].

In peripheral ameloblastoma, the stellate reticulum is seldom conspicuous and most of the epithelial islands exhibit palisading of columnar basal cells. Bone or periosteum is not involved, though the tumor may produce a superficial impression on the underlying bone.

Metastasizing ameloblastoma also has the typical well-differentiated benign histology of the solid/multicystic type at the primary site, but in addition, similar foci of benign histology are also seen in locations remote from the primary site, considered to be metastases [29]. Since histological differentiation from non-metastasizing ameloblastoma is not possible, the diagnosis of metastasizing ameloblastoma can be made only retrospectively, when metastasis has occurred. Follicular ameloblastoma was most frequently encountered at the secondary site followed by the plexiform type. Lungs were the most commonly affected secondary sites [27].

Staging

Based on clinicopathological features, Yang et al. staged ameloblastomas into three stages: stage I, the maximum tumor diameter ≤6 cm; stage II, the maximum diameter of tumor >6 cm or tumor invasion into the maxillary sinus or orbital floor; and stage III, tumor invasion of the skull base or metastasis into regional lymph nodes [32]. There is a significant relationship between time to recurrence and tumor stage, with the earliest recurrence in stage III tumors [32].

Treatment

The treatment modality of choice for ameloblastoma is surgery. Other modalities like chemotherapy or radiotherapy have a limited role and only in select situations.

Surgery

The goal of surgical treatment of ameloblastomas is to minimize recurrences and restore good function and aesthetics with minimum morbidity in the donor area. The currently recommended surgery for classic ameloblastoma (solid/multicystic type) is complete en bloc resection (radical surgery) with an adequate margin of safety, which is classified as segmental or marginal osteotomy for the mandible and partial or total maxillectomy for the maxilla. Due to the high recurrence rate after conservative surgery, particularly for solid/multicystic ameloblastomas, a wide resection with a 1 to 1.5 cm bony margin is recommended. However, a recent meta-analysis could not prove the superiority of radical surgery over conservative surgery [33]. Recurrence is attributed to the infiltration of tumor cells into the cancellous bone beyond the periphery of radiographic margins [34]. Immediate or delayed bone reconstruction and dental rehabilitation have to be ensured to help with speech and swallowing and improve patient outcomes [29].

However, radical surgery leads to aesthetic deformities, functional impairments, and psychological distress. To avoid these complications, conservative surgery has also been tried, which includes marsupialization, enucleation, curettage, enucleation combined with Carnoy’s solution, enucleation combined with curettage, and curettage combined with cryotherapy. However, these conservative approaches lead to a high recurrence rate, which has been reported to be 40% in a recent meta-analysis [35]. For treatment of primary solid/multicystic ameloblastoma when conservative treatment was performed, the relative risk of recurrence was three-fold higher in comparison to radical treatment [35]. Similarly in another meta-analysis that included four studies on radical versus conservative treatment approach for ameloblastoma, a higher recurrence rate after a conservative approach compared to the surgical approach was documented [36]. Thus, the treatment of primary multicystic ameloblastoma with bone resection is justified.

For mandibular reconstruction, vascularized free bone grafts (from the fibula, ilium, scapula, or radius) are the standard; the flap of choice is the fibular free flap, which has the added advantage of reconstructing long segment mandibular defects [37].

The iliac crest-internal oblique free flap is particularly well suited for mandibular reconstruction because of the utility of the internal oblique muscle in the formation of a soft tissue closure over the bone. For mandibular angle defects, the iliac crest is favored, eliminating the need for multiple osteotomies as seen with the fibula [29]. Those patients who are reconstructed with an osteomyocutaneous free flap may be considered for osteointegrated dental implants [38]. Pappalardo et al. suggest that osseointegrated implants lead to significantly better masticatory function, fewer psychological consequences, and improved quality of life [39]. Distraction osteogenesis is also finding increased use in mandibular reconstruction following surgical resection of the tumor [40]. The recent use of virtual 3D surgical planning has further improved outcomes by making reconstructive surgery much more predictable and precise [41].

Reconstruction of maxillary defect is done using a skin graft to line the cavity. An obturator is fitted thus allowing easy access to the resection bed during surveillance. The cortical bone of the maxilla offers less resistance to tumor invasion as compared to the mandible and therefore ameloblastoma of the maxilla has a higher local recurrence rate after a surgical procedure. Hence, free flaps are not used for maxillary defects to avoid covering a potential recurrence site [29].

For unicystic ameloblastomas, both the radical as well as the conservative surgical approach, including excision, marsupialization, chemical electrocautery, curettage, radiation therapy, or combined surgery and radiation, may be employed [1,29,42]. The extraosseous/peripheral ameloblastoma is mostly treated with wide local excision, and a recurrence rate of 9%-20% following treatment has been reported [1].

Non-surgical treatment

Systemic chemotherapy has no role in the treatment of localized ameloblastoma, however, in metastatic settings, chemotherapy remains the only choice of treatment. Numerous agents and combinations have been employed using cyclophosphamide, methotrexate, 5-fluorouracil, vinblastine, cisplatin, bleomycin, adriamycin, paclitaxel-carboplatin, doxorubicin, and gemcitabine. Ameloblastoma may be sensitive to platinum-based anticancer agents.

Recently, with the elucidation of molecular markers of ameloblastoma, there have been attempts for the treatment of ameloblastoma with molecular targeted therapy. These tumors have highly recurrent somatic mutations in the signaling pathways of mitogen-activated protein kinase (MAPK) and sonic hedgehog (SHH), which are known to be activated during tooth development. Within the MAPK pathway, the BRAFV600E mutation is found in 57% of ameloblastomas, while within the SHH pathway, Smoothened (SMO) mutations have been identified in 24% of ameloblastomas [6,29]. Interestingly, ameloblastomas with BRAFV600E mutations are predominantly (96%) located in the mandible and ameloblastomas with SMO mutations are predominantly found in the maxilla (85%). Molecular targeted therapy drugs that have the potential to be used in ameloblastoma are those which inhibit the functions of mutated BRAF and MEK. The US Food and Drug Administration (FDA) has approved three molecular targeted therapies for the BRAF V600E mutation: vemurafenib and dabrafenib for BRAF mutation and trametinib for MEK mutation [43]. In a recent study, multiple recurrent ameloblastomata in the mandible with metastatic masses in both lungs responded dramatically to therapy with a dual BRAF/MEK inhibition by dabrafenib/trametinib [44]. Similarly, another report showed a notable reduction in tumor volume in a multiply recurrent ameloblastoma of the mandible in response to single BRAF inhibition therapy with dabrafenib [45].

Radiotherapy

Similar to chemotherapy, radiotherapy also has a limited role in the management of ameloblastoma. It may be utilized in patients with post-surgical microscopic or gross residual disease, poor surgical candidates, or those with disease not amenable to re-resection [38]. Dose-fractionation schedules needed for tumor control are similar to those used for carcinomas and range from 66 Gy in 33 once-daily fractions five fractions per week for microscopic residual disease to 70 Gy in 35 once-daily fractions for a treatment duration of seven weeks [38]. In a recent pooled analysis of three studies on radiotherapy for ameloblastoma, local tumor control was achieved in seven of nine (78%) patients irradiated for gross disease and three of three (100%) patients treated for microscopic residual disease after surgery [23]. Newer technologies for radiotherapy such as image-guided radiotherapy, stereotactic radiotherapy, intensity-modulated radiotherapy, and proton beam therapy may be beneficial for patients with extensive maxillary ameloblastomas extending to the skull base to effectively treat the tumor without significant dose to the CNS and visual apparatus [38].

Prognosis

The prognosis for ameloblastoma depends on the age of the patient, location and size of the tumor, histological type, extent, and stage of disease [29]. Ameloblastoma is a slow-growing tumor and a meta-analysis calculated the mean specific growth rate of ameloblastoma to be 87.8% per year [28]. However, if left untreated, ameloblastoma can grow to a huge size and pose a risk to the airway [46].

After treatment, ameloblastoma may recur with an overall recurrence rate of 9.8% according to a Chinese study [32] and 19.3% as per a European multicenter study [24]. Recurrence rates are dictated by the type of surgery used (radical versus conservative), the adequacy of the surgical margins, and in cases of maxillary ameloblastoma extension into vital structures such as the orbit, paranasal sinuses, or skull base. Recurrence following conservative surgery is due to the persistence of residual disease, which grows slowly within the evacuated cavity while radical surgical resection shows far lesser recurrence rates. More than 50% of recurrences occur within five years of the primary surgical intervention [47]. Tumors that are larger than 6 cm in size or involve adjacent anatomical structures, including soft tissue are associated with a higher recurrence rate irrespective of the type of surgery [32]. A higher recurrence rate is also reported in granular and follicular histological subtypes [47]. Generally, maxillary ameloblastomas are more aggressive and prone to higher recurrence than mandibular, mainly due to the thin maxillary cortical bone that provides a weak barrier for the local spread of the tumor.

Death may occur in patients with untreated maxillary ameloblastoma extending into the central nervous system or in patients having multiple recurrences.

Recent advances

In the last decade, many exciting new developments have taken place that have increased our understanding of the pathogenesis of ameloblastoma, which has translated into newer treatment options. The first of these is the detection of a high incidence of BRAF V600E and SMO L412F mutations in ameloblastoma [6,48-49]. The oncogenic BRAFV600E mutation leads to the activation of the mitogen-activated protein kinase (MAPK) pathway, which has resulted in successful treatment with a BRAF inhibitor. The expression of ADP-ribosylation factor (ARF)-like 4c (ARL4C), induced by a combination of the EGF-MAPK pathway and Wnt/β-catenin signaling, has been shown to induce epithelial morphogenesis. Overexpression of ARL4C, due to alterations in the EGF/RAS-MAPK pathway and Wnt/β-catenin signaling, promotes tumorigenesis. The RAF1-MEK/ERK-ARL4C axis may function in cooperation with the BRAFV600E-MEK/ERK pathway to promote ameloblastoma development [50]. The microRNA miR-29a-3p promotes migration and invasion in ameloblastoma via Wnt/β-catenin signaling by targeting catenin beta interacting protein 1 [51]. The upfront treatment with BRAF inhibitors resulting in substantial tumor regression has enabled non-mutilating complete surgical removal, ad integrum bone regeneration, and organ preservation. Most patients show a marked radiologic and clinical response to medical treatment, enabling successful conservative surgery. Face preservation therapy could be achieved in pediatric patients presenting with BRAF V600E mutated ameloblastoma [52].

The second development is the understanding of the biological factors that cause the invasive nature of ameloblastoma. In addition to the BRAFV600E mutation, ameloblastomas were also found to harbor dysregulated tumor suppressor genes such as p53 and oncogenes like BCL2. Other cancer-related genes, such as IGF2 and matrix metalloproteinases (MMPs), are also among dysregulated genes in ameloblastoma [53]. There is evidence to indicate that tumor cells secrete factors related to bone resorption and invasion, such as MMPs and receptor activator nuclear factor kappa B ligand (RANKL), and interact with surrounding stromal components to construct a microenvironment favorable for tumor growth in ameloblastoma [54]. In addition, there is reduced activation of immune reaction in the ameloblastoma tumor microenvironment. Karpathiou et al. have found that PD-L1 expression was seen in almost half of the tumors and that CD8+ T cells and M2 macrophages are found less in number [55].

Among the recently developed surgical approaches for the treatment of ameloblastoma, two merits are worth mentioning. The recent use of virtual 3D surgical planning has improved outcomes by making reconstructive surgery much more predictable and precise [41]. Recent studies have shown that the use of computer-assisted surgery (CAS) and cutting guides based on predetermined surgical margins do not compromise the margin status in surgical resections of ameloblastoma and could potentially decrease the occurrence of close or positive margins [56]. Recently, active decompression with distraction sugosteogenesis (ADDS) has been successfully used for the treatment of conventional ameloblastoma. Till now, ADDS use was limited to the conservative management of odontogenic cystic conditions. Wiscovitch et al. showed that ADDS can prove to be a viable treatment because it demonstrates a reduction in the size of the initial lesion by new osseous formation within two weeks of placement of the device [57].

After successful surgery for ameloblastoma, prevention of recurrence has been a long-standing issue. Recently, a machine learning algorithm has been used to predict recurrence with reliable accuracy. The four most important variables influencing ameloblastoma recurrence were the time elapsed from treatment, initial surgical treatment, tumor size, and radiographic presentation. These were used in the algorithm to reliably predict recurrence [58]. Similarly, Yang et al. have developed a favorable nomogram that accurately predicted the recurrence-free survival of patients with ameloblastoma based on individual characteristics, which could optimize tailored therapy and follow-up [59].

## Conclusions

Ameloblastoma is the most commonly occurring odontogenic tumor of the mandible and maxilla. It is a locally invasive but slow-growing tumor that presents with painless swelling of the mandible or maxilla. The best treatment of ameloblastoma is aggressive en bloc resection with simultaneous reconstruction. The high recurrence rate and large tissue defects have been long-standing issues in treating ameloblastomas. Recent molecular developments strongly suggest the possibility of targeted therapy with better outcomes in ameloblastomas.
